# Breastfeeding Outcomes After Scheduled Cesarean Section Under an ERAS Pathway: An Analytical Observational Study

**DOI:** 10.3390/nursrep16040134

**Published:** 2026-04-13

**Authors:** Salomé Moreno-Vega, José C. Vilches, Francisco García-Pedrajas, Isabel María Morales-Gil, Cristóbal Rengel-Díaz

**Affiliations:** 1 Department of Obstetrics and Gynecology, Hospital Quirón Salud Málaga, 29004 Málaga, Spain; 2 Faculty of Health Sciences, University of Málaga, 29010 Málaga, Spain; 3 Department of Anesthesiology and Reanimation, Hospital Quirón Salud Málaga, 29004 Málaga, Spain; 4 Andalusian Health Service, 29740 Málaga, Spain

**Keywords:** ERAS, cesarean section, skin-to-skin contact, breastfeeding, women-centered care, LATCH, postoperative recovery

## Abstract

**Background/Objectives:** Breastfeeding initiation after cesarean section is frequently delayed due to postoperative routines and early mother–infant separation. Enhanced Recovery After Surgery (ERAS) protocols have been introduced in obstetrics to improve maternal recovery and may facilitate practices aligned with a family-centered model of care. The aim of this study was to evaluate the association between ERAS implementation and breastfeeding outcomes, including early feeding patterns and effective breastfeeding at discharge. **Methods:** An analytical longitudinal study was conducted including women undergoing scheduled cesarean section between January 2025 and November 2025 at Quirón Salud Málaga Hospital (Spain). A total of 131 women were enrolled in this study. Two groups were compared: an exposed group that received an ERAS protocol (*n* = 65) for scheduled cesarean section and a control group (*n* = 66) managed with conventional in-hospital care. An intrasubject analysis was conducted, and associations were assessed using odds ratios (ORs) with 95% confidence intervals (CIs). Multivariable logistic regression was performed to identify factors independently associated with effective breastfeeding. **Results:** The ERAS group showed a stable feeding pattern over time, with a high persistence of exclusive breastfeeding (Stuart–Maxwell χ^2^(2) = 1.14; *p* = 0.565). In multivariable analysis, ERAS implementation remained an independent factor (adjusted OR 3.79; 95% CI 1.50–9.55; *p* = 0.005), together with early skin-to-skin (adjusted OR 2.68; 95% CI 1.13–6.36; *p* = 0.026), as was breastfeeding support (adjusted OR 2.72; 95% CI 1.02–7.22; *p* = 0.045). LATCH scores were also higher in the ERAS group (*p* = 0.0005; r = 0.34). **Conclusions:** Women managed under ERAS presented a higher prevalence of exclusive breastfeeding at hospital discharge and better breastfeeding performance. ERAS implementation was associated with improved breastfeeding outcomes, possibly through clinical conditions that facilitate early contact and structured breastfeeding support.

## 1. Introduction

The cesarean section is one of the most frequently performed surgical procedures worldwide. Although often necessary for maternal or fetal indications, it is associated with significant challenges, including acute postoperative pain, delayed maternal recovery, and difficulties in establishing breastfeeding. Compared with vaginal birth, women undergoing cesarean section experience higher rates of delayed breastfeeding initiation and lower rates of exclusive breastfeeding [1].

Early mother–infant skin-to-skin contact during the “golden hour” after birth is a critical evidence-based strategy to promote breastfeeding initiation, neonatal thermoregulation, and early bonding [2,3]. Despite these well-documented benefits, early skin-to-skin contact has historically been limited in the surgical setting due to operating room routines and safety concerns, frequently resulting in the avoidable separation of the mother–newborn dyad [3].

Lately, maternity care has shifted toward a woman-centered and respectful approach. This paradigm emphasizes dignity and maternal autonomy by integrating evidence-based clinical practices with the emotional and social dimensions of childbirth [2,3]. Within this framework, Enhanced Recovery After Surgery (ERAS) protocols have been introduced in obstetrics as a structured approach to optimize postoperative recovery [4,5].

ERAS Society guidelines provide standardized recommendations across the antenatal, intraoperative, and postoperative periods. Their implementation has demonstrated significant benefits in reducing postoperative pain, shortening hospital stays, and accelerating maternal functional recovery [6,7,8]. While multiple studies have confirmed these clinical advantages in elective cesarean section [9,10,11], ERAS pathways may also serve as a facilitator for woman-centered care by reducing clinical barriers to early mother–infant interaction.

Nonetheless, despite the growing evidence supporting ERAS in obstetrics, data regarding its specific impact on breastfeeding-related outcomes remain limited and inconsistent. Therefore, the aim of this study was to evaluate the association between ERAS implementation and breastfeeding outcomes, including early feeding patterns and effective breastfeeding at discharge.

## 2. Materials and Methods

### 2.1. Study Design and Participants

An analytical prospective observational, non-randomized study was conducted to evaluate the impact of an ERAS protocol on breastfeeding. This study adheres to the Strengthening the Reporting of Observational Studies in Epidemiology (STROBE) guidelines, and the corresponding checklist is provided as Supplementary Table S1.

Women managed under the ERAS pathway constituted the exposed group, while the comparison group consisted of women receiving conventional perioperative care, reflecting standard practice prior to ERAS implementation. Both care models coexisted during the study period as part of routine clinical practice. Patients were not randomized or selected based on clinical characteristics. Patients were consecutively scheduled for cesarean section according to routine clinical practice. Allocation to ERAS or standard care was not randomized and did not depend on patient clinical characteristics but rather on the organizational context in which care was delivered at the time of surgery. The ERAS pathway was implemented by a multidisciplinary team of trained midwives, obstetricians, anesthesiologists, and nursing staff, ensuring standardized protocol delivery. The objective of this design was to evaluate whether implementation of the ERAS protocol provided measurable clinical benefits that would support its adoption as the standard care pathway for scheduled cesarean section.

### 2.2. Study Setting and Population

This study was conducted at Quirónsalud Málaga Hospital (Spain). The exposed group, managed under the ERAS protocol, included 65 women undergoing scheduled cesarean section. The non-exposed (control) group, receiving the conventional care protocol, consisted of 66 women undergoing scheduled cesarean section. Sample size was justified based on the comparison of two proportions for the primary outcome, effective breastfeeding at discharge. Assuming rates of 50% in the standard care group and 75% in the ERAS group, with a two-sided alpha of 0.05 and 80% power, 116 women were required. After allowing for 10% attrition, the estimated target sample size was 128 women. The final sample of 131 women therefore exceeded this requirement.

### 2.3. Inclusion and Exclusion Criteria

This study included women with an indication for a scheduled cesarean section, singleton pregnancies with no evidence of fetal malformations or relevant fetal pathology, and age ≥ 18 years. Exclusion criteria were: (a) admission to the Intensive Care Unit (ICU) following cesarean section; (b) withdrawal of informed consent at any time; and (c) language barriers preventing an adequate understanding of the study information or the provision of informed consent.

### 2.4. Exposure

The ERAS pathway implemented in this study represents a structured perioperative care model aimed at optimizing maternal recovery after scheduled cesarean section and facilitating early mother–infant interaction. The program was delivered by a multidisciplinary team comprising midwives, obstetricians, anesthesiologists, and nursing staff who had received specific training in ERAS principles. The ERAS protocol included evidence-based interventions applied during the preoperative, intraoperative, and immediate postoperative periods, such as optimized fasting, regional anesthesia, multimodal analgesia, early mobilization, and early oral intake. The ERAS protocol included specific components aimed at promoting breastfeeding and early maternal–neonatal interaction, such as early and sustained skin-to-skin contact, encouragement of rooming-in, avoidance of unnecessary mother–infant separation, and proactive lactation support provided by trained midwives. Skin-to-skin contact was only interrupted in cases of maternal or neonatal instability requiring medical intervention, and routine supplementation was not implemented unless clinically indicated.

A detailed description of ERAS components related to breastfeeding, including their implementation and measurement, is provided in Supplementary Table S2.

Standardized care pathways and predefined clinical criteria guided the application of ERAS measures to ensure consistency in care delivery. The ERAS pathway applied in this study was based on the ERAS Society recommendations for cesarean delivery, which address modifiable preoperative, intraoperative, and postoperative clinical factors to optimize maternal recovery and perioperative care [7].

### 2.5. Variables

Sociodemographic and obstetric variables included maternal age, parity, previous cesarean section, and prenatal intention to breastfeed, nationality, and educational level. Early skin-to-skin contact was defined as direct mother–infant contact between the mother’s chest and the newborn, with no clothing or barriers between them, except for a covering placed over the dyad to maintain thermal regulation. It was initiated as soon as clinically feasible, either in the operating room or in the recovery room, and maintained during the immediate postoperative period, covering at least the first 2 h after birth unless interrupted due to maternal or neonatal instability requiring medical intervention. Neonatal characteristics, including birth weight and umbilical arterial cord pH, were also recorded. Breastfeeding outcomes constituted the primary study variables. Breastfeeding support in the recovery room was defined as active assistance provided by a midwife to facilitate breastfeeding initiation, including positioning and latch support during the immediate postoperative period. This support was provided during the stay in the recovery room (approximately the first 2 h after cesarean section) and recorded as a binary variable (yes/no) based on routine clinical documentation. Although midwives were not formally IBCLC-certified, they had substantial clinical experience in breastfeeding support and were trained in ERAS principles. Effective breastfeeding at discharge was defined as a composite outcome combining exclusive breastfeeding at discharge and a LATCH score ≥ 7. Exclusive breastfeeding was defined as feeding exclusively with breast milk at the time of hospital discharge. Breastfeeding effectiveness was assessed using the LATCH scoring system, a validated tool available in Spanish [12], with a reported Cronbach’s alpha value of 0.95, indicating high reliability. This tool evaluates latch quality, audible swallowing, nipple type, maternal comfort, and the need for assistance. Scores range from 0 to 10, with higher scores indicating more effective breastfeeding. The LATCH score reflects the effectiveness of breastfeeding technique at a specific point in time, whereas exclusive breastfeeding represents a feeding outcome at discharge. In our study, both variables were intentionally included to capture different but related dimensions of breastfeeding.

Other feeding types, including mixed and formula feeding, were also recorded. Maternal variables such as age, parity, previous cesarean section, and prenatal intention to breastfeed were considered potential confounders based on clinical relevance and the existing literature.

### 2.6. Data Collection

This study was conducted from January 2025 to November 2025. Data were collected prospectively during the hospital stay using standardized clinical records. Maternal variables were recorded on the day of the cesarean section as part of routine clinical assessment.

Breastfeeding effectiveness during hospitalization was assessed using the LATCH scoring system, recorded by trained midwives in a dedicated data collection form prior to hospital discharge.

Feeding type was assessed at two predefined time points: during the immediate postoperative period (in the recovery room) and at hospital discharge. The primary outcome, effective breastfeeding at discharge, was defined based on feeding status and LATCH score at the time of discharge and obtained from hospital records. Postpartum follow-up visits were conducted as part of routine care; however, these were not used to define the primary outcome of this study.

### 2.7. Statistical Analysis

Quantitative variables were expressed as means and standard deviations (SD), while qualitative variables were described as frequencies and percentages. Feeding type (categorized as exclusive breastfeeding, mixed feeding, or formula feeding) was assessed at two time points: during the immediate postoperative recovery period and at hospital discharge. To evaluate within-subject changes in feeding patterns over time within each study group, an intrasubject analysis was performed using the Stuart–Maxwell test for marginal homogeneity, which is appropriate for paired categorical data with more than two categories. Statistical significance was set at *p* < 0.05.

Comparisons between ERAS and control groups were performed using the chi-square test or Fisher’s exact test for categorical variables, and the Mann–Whitney U test for non-normally distributed continuous variables, as appropriate. To quantify the clinical relevance of the observed differences, effect size (r) was calculated from the standardized Z statistic derived from the Mann–Whitney test using the formula r = Z/√N, where N corresponds to the total number of participants. The magnitude of the effect size was interpreted according to Cohen’s conventional thresholds.

A multivariable logistic regression analysis was performed to identify factors independently associated with effective breastfeeding at discharge, including early skin-to-skin contact, prenatal intention to breastfeed, parity, previous cesarean section, maternal age, maternal educational level, breastfeeding support in the recovery room, ERAS group, and neonatal ICU admission. Educational level was entered as a categorical variable (university education vs. non-university education). In addition to early skin-to-skin contact and breastfeeding support, the ERAS pathway included spinal anesthesia combined with transversus abdominis plane (TAP) block, early mobilization (<6 h), early urinary catheter removal, and early oral tolerance (<4 h). These measures were considered intrinsic components of the ERAS intervention and were therefore not included as independent variables in the regression analysis to avoid overadjustment. Variables included in the multivariable regression models were selected a priori based on clinical relevance and the existing literature. Model fit was assessed using the Hosmer–Lemeshow goodness-of-fit test, and Nagelkerke’s R^2^ was used to describe the variance explained by the model. Results are presented as adjusted ORs with 95% CI and *p*-values. Multicollinearity was evaluated using variance inflation factors (VIF), with all values below 2. The number of events per variable was considered to reduce the risk of overfitting. All statistical analyses were performed using SPSS software (version 28.0; IBM Corp., Armonk, NY, USA).

### 2.8. Ethical Considerations

This study was approved by the Malaga Provincial Research Ethics Committee (approval code: SICEIA-2024-001667; approval date: 31 January 2025). Written informed consent was obtained from all participants prior to the cesarean section. The research was conducted in accordance with the Declaration of Helsinki (1975, revised 2013) and complied with Spanish Organic Law 3/2018 on the Protection of Personal Data and Guarantee of Digital Rights. Confidentiality and anonymity were rigorously maintained.

## 3. Results

### 3.1. Baseline Maternal and Neonatal Characteristics

A total of 131 women undergoing scheduled cesarean section were included in this study, of whom 66 (50.4%) received conventional care and 65 (49.6%) were managed under an ERAS pathway. The mean maternal age was 35.9 ± 5.4 years, and the mean gestational age at delivery was 38.4 ± 1.0 weeks. The study population was predominantly of Spanish nationality (98.0%), and more than half of the participants (54.2%) had university-level education. Regarding obstetric history, 46.6% of the women had a history of previous cesarean section, and the majority were multiparous. The mean birth weight was 3172.6 ± 491.5 g, and the mean umbilical arterial pH at birth was 7.258 ± 0.04. Although a slight difference was observed in gestational age at delivery (*p* = 0.010), baseline maternal and neonatal characteristics were generally comparable between the ERAS and control groups, with no clinically relevant differences observed (Table 1).

### 3.2. ERAS Protocol and Breastfeeding Outcomes

A longitudinal analysis of feeding types was conducted to evaluate clinical progression from the immediate postoperative period to hospital discharge within each study group. Feeding patterns were assessed during recovery and discharge, with intrasubject changes analyzed using the Stuart–Maxwell test for marginal homogeneity (Table 2).

In the control group (*n* = 66), the distribution of feeding types showed a shift toward mixed feeding between the immediate postoperative period and discharge. Although this change approached statistical significance, it did not reach the predefined threshold (Stuart–Maxwell χ^2^(2) = 5.47; *p* = 0.065). Specifically, the exclusive breastfeeding rates decreased from 43.9% to 37.8%, while mixed feeding increased from 43.9% to 48.4%.

In contrast, the ERAS group (*n* = 65) exhibited a stable feeding pattern throughout the hospital stay, with no significant intrasubject changes observed (Stuart–Maxwell χ^2^(2) = 1.14; *p* = 0.565). This group maintained high rates of exclusive breastfeeding from the immediate recovery period (75.8%) through hospital discharge (71.3%). When comparing both groups, exclusive breastfeeding at discharge was more frequent in the ERAS group (71.3% vs. 37.8%; *p* < 0.001).

### 3.3. Quality of Breastfeeding Technique

Regarding the quality of the breastfeeding technique at discharge, LATCH scores were significantly higher in the ERAS group (Median = 8; IQR 7–9) compared to the control group (Median = 7; IQR 5–8; *p* = 0.0005), as shown in Table 3. This difference represented a moderate effect size (r = 0.34), indicating a clinically relevant improvement in latch quality associated with the ERAS pathway.

### 3.4. Factors Associated with Effective Breastfeeding at Discharge

The rate of effective breastfeeding, defined as the combination of exclusive breastfeeding and a LATCH score ≥ 7 at discharge, was significantly higher in the ERAS group compared to the control group (72.9% vs. 42.3%; OR = 3.67; 95% CI: 1.58–8.82; *p* = 0.0025). Regarding protocol adherence, early skin-to-skin contact was also significantly more frequent in the ERAS pathway compared to the control group (66.7% vs. 30.8%; OR 4.50; 95% CI 2.16–9.38; *p* < 0.001).

To identify the independent predictors associated with effective breastfeeding at discharge, a multivariable logistic regression analysis was performed, as shown in Table 4. ERAS implementation remained independently associated with a significantly higher likelihood of effective breastfeeding at discharge (adjusted OR 3.79; 95% CI 1.50–9.55; *p* = 0.005). Early skin-to-skin contact was also independently associated with effective breastfeeding (adjusted OR 2.68; 95% CI 1.13–6.36; *p* = 0.026), as was breastfeeding support during the immediate postoperative period (adjusted OR 2.72; 95% CI 1.02–7.22; *p* = 0.045). Maternal age, educational level, parity, previous cesarean section, prenatal intention to breastfeed, and neonatal intensive care unit admission were not significantly associated with breastfeeding effectiveness in the adjusted model (*p* > 0.05). The multivariable model showed good calibration (Hosmer–Lemeshow χ^2^(8) = 13.42; *p* = 0.098) and explained 34.5% of the variance in effective breastfeeding at discharge (Nagelkerke R^2^ = 0.345).

## 4. Discussion

The present study suggests that the implementation of an ERAS pathway is associated with more favorable breastfeeding outcomes after scheduled cesarean section. Women managed under ERAS showed higher rates of exclusive breastfeeding at discharge, higher LATCH scores, and a lower proportion of mixed feeding compared with women receiving conventional care. These findings extend the existing literature on ERAS beyond maternal recovery outcomes such as pain control, mobilization, and length of hospital stay [6,7,8,9,10,11].

Our findings suggest that the association between ERAS and breastfeeding outcomes is unlikely to reflect a direct biological mechanism. Instead, this relationship may be explained by the clinical and organizational conditions promoted by ERAS pathways, which facilitate early mother–infant interaction and structured breastfeeding support. This interpretation is consistent with existing evidence highlighting the role of early skin-to-skin contact and continuous maternal support in breastfeeding initiation and effectiveness [2,3]. Although educational level was included in the multivariable model, it was not independently associated with effective breastfeeding at discharge, suggesting that immediate postpartum practices and early mother–infant interaction may play a more relevant role than sociodemographic characteristics alone. Within this framework, ERAS may function as an enabling care pathway that reduces organizational and clinical barriers to practices known to promote breastfeeding success.

The longitudinal analysis provides further insight into feeding patterns over time. Among women receiving conventional care, feeding practices showed a tendency to change from the immediate postoperative recovery period to hospital discharge, whereas feeding patterns in the ERAS group remained more stable. Although these intrasubject changes were not statistically significant, the observed stability in the ERAS group may reflect a more consistent pattern of breastfeeding during hospitalization.

Breastfeeding effectiveness, assessed using the LATCH scoring system, was higher in the ERAS group at discharge. This finding is consistent with a more favorable breastfeeding performance, including latch quality and maternal-infant interaction. The use of the LATCH score provides additional clinically relevant information beyond feeding status alone. While exclusive breastfeeding reflects the type of feeding at discharge, the LATCH score evaluates the effectiveness of breastfeeding technique. Previous studies have shown that higher LATCH scores in the early postpartum period are associated with improved breastfeeding continuation and duration, whereas lower scores may identify mother–infant dyads at risk of early breastfeeding difficulties [13,14,15,16,17]. Therefore, these measures should be interpreted as complementary rather than interchangeable. In this context, the composite definition of effective breastfeeding used in this study, combining exclusive breastfeeding with a LATCH score ≥ 7, allows for a more comprehensive assessment of breastfeeding success at discharge.

The association observed in our study between ERAS implementation and effective breastfeeding is consistent with ERAS Society recommendations, which advocate for intraoperative adaptations that facilitate early mother–infant contact during cesarean delivery when clinically feasible [18]. Although several studies have reported positive associations between ERAS pathways and breastfeeding outcomes, the available evidence remains heterogeneous. Previous systematic reviews have highlighted variability in both study design and reported outcomes, with some studies showing improvements in breastfeeding-related outcomes and others reporting no significant differences [5,9,10]. In addition, the overall quality and consistency of evidence regarding breastfeeding outcomes within ERAS protocols remain limited, as most studies have primarily focused on maternal recovery indicators rather than feeding outcomes [9,10]. These discrepancies may be explained by differences in ERAS implementation, local clinical protocols, and the extent to which breastfeeding-supportive practices are integrated into care pathways. This suggests that the impact of ERAS on breastfeeding outcomes may be context-dependent rather than uniform across settings.

Structured cesarean care models incorporating perioperative planning and humanized surgical adaptations have previously reported higher rates of skin-to-skin contact and improved early breastfeeding indicators [19]. In our study, early skin-to-skin contact was significantly more frequent in the ERAS group and remained independently associated with effective breastfeeding after adjustment for maternal and neonatal variables. These findings are consistent with the hypothesis that system-level organizational factors may influence early feeding success beyond individual maternal characteristics.

Furthermore, a systematic review evaluating early skin-to-skin contact after cesarean section consistently reported improvements in breastfeeding initiation, latch quality, and maternal satisfaction [20]. Our results align with this body of evidence and provide prospective data suggesting that ERAS pathways may serve as an operational framework through which these evidence-based practices can be consistently implemented in routine clinical care.

Emerging evidence also suggests that early mother–infant contact may have implications beyond feeding outcomes, potentially influencing neonatal physiological adaptation and early microbial colonization [21]. Although these aspects were not evaluated in the present study, they further support the potential relevance of promoting early contact after cesarean section when clinically feasible.

Some limitations of this study should be acknowledged. Group allocation was not randomized, as random assignment to ERAS or conventional care was not feasible during the initial phase of ERAS implementation in our institution. Due to the study design, randomization and blinding were not possible, which may introduce residual confounding.

This study was conducted in a single center with a relatively homogeneous population and standardized clinical protocols. Therefore, the findings may not be directly generalizable to other settings with different patient populations, healthcare structures, or ERAS implementation strategies. However, the use of standardized clinical protocols may enhance internal validity and reduce variability in care, allowing for a clearer assessment of the associations observed.

In addition, relevant factors, such as previous breastfeeding experience and social support, were not systematically collected and could not be included in the analysis.

Despite these limitations, the present prospective comparative study provides novel and clinically relevant evidence in an area where data remain limited. While most ERAS research in elective cesarean section has focused on maternal recovery outcomes, evidence regarding breastfeeding-related outcomes is scarce. By showing higher rates of effective and exclusive breastfeeding at discharge associated with ERAS-facilitated early skin-to-skin contact, this study highlights the broader potential impact of ERAS pathways beyond maternal recovery alone, including their relevance for midwifery practice.

Although the present study focused on scheduled cesarean sections in low-risk pregnancies, future research should explore the applicability and impact of ERAS pathways in more complex obstetric scenarios. In particular, the potential benefits of ERAS implementation in urgent or emergency cesarean sections remain insufficiently studied. These situations often involve higher maternal and neonatal vulnerability, where optimized recovery strategies could be especially relevant.

## 5. Conclusions

This study found that, in women undergoing scheduled cesarean section, the implementation of an ERAS pathway was associated with more favorable breastfeeding outcomes. Women managed under ERAS presented a higher prevalence of exclusive breastfeeding at hospital discharge and higher breastfeeding performance, as assessed by LATCH scores, compared with women receiving conventional care.

Breastfeeding support during the immediate postoperative period was also independently associated with effective breastfeeding, whereas parity, previous cesarean section, and maternal age were not associated with breastfeeding outcomes in this cohort.

From a longitudinal perspective, feeding patterns in the ERAS group remained stable from the immediate postoperative recovery period to hospital discharge, while greater variability was observed among women receiving conventional care. This finding may reflect a more consistent pattern of breastfeeding during hospitalization among women managed under an ERAS pathway.

Overall, these findings highlight an association between ERAS implementation, early skin-to-skin contact, and effective breastfeeding after scheduled cesarean section, although causality cannot be inferred given the observational design of this study.

## Figures and Tables

**Table 1:** Baseline maternal and neonatal characteristics of the study population (*N* = 131).

Variable	Unit	Control Group (*n* = 66) Mean ± SD/%	ERAS Group (*n* = 65) Mean ± SD/%	*p*-Value *
Maternal characteristics				
Maternal Age	Years	36.2 ± 5.5	35.6 ± 5.3	0.440
Weight	kg	78.9 ± 14.4	76.5 ± 12.8	0.303
Height	Cm	165 ± 5	167 ± 5	0.056
Body mass index	kg/m^2^	29.1 ± 4.2	28.9 ± 3.8	0.680
Gestational age at delivery	Weeks	38.21 ± 1.04 (95% CI 37.96–38.46)	38.65 ± 0.84 (95% CI 38.45–38.85)	0.010
Previous cesarean section	Yes	55.8%	37.5%	0.097
	No	44.2%	62.5%	
Parity	Primiparous	32.7%	47.9%	0.220
	Secundiparous	36.5%	37.5%	
	Multiparous (≥3)	23.1%	10.4%	
Prenatal intention to breastfeed	Yes	89.2%	89.4%	0.970
	No	10.8%	10.6%	
Nationality	Spanish	97.9%	98.1%	0.360
	Other	2.1%	1.9%	
Educational level	Secondary/vocational	46.3%	43.9%	0.574
	University	52.7%	54.2%	
	Postgraduate	1.0%	1.9%	
Neonatal characteristics				
Birth weight	g	3238.6 ± 559.6	3105.8 ± 402.2	0.121
Umbilical arterial pH	–	7.258 ± 0.05	7.259 ± 0.02	0.831

SD: Standard deviation. * *p*-values are calculated using Student’s *t*-test or Mann–Whitney U test for continuous variables and chi-square or Fisher’s exact test for categorical variables, as appropriate.

**Table 2:** Feeding type during hospitalization according to study group (*n* = 131).

Study Group	Feeding Type	Recovery, *n* (%)	Discharge, *n* (%)	*p*-Value *
Control Group (*n* = 66)	Exclusive breastfeeding	29 (43.9)	25 (37.8)	0.065
	Mixed feeding	29 (43.9)	32 (48.4)	
	Formula feeding	8 (12.1)	9 (13.6)	
ERAS Group (*n* = 65)	Exclusive breastfeeding	49 (75.8)	47 (71.3)	0.565
	Mixed feeding	9 (13.8)	10 (15.3)	
	Formula feeding	7 (10.7)	8 (12.3)	

* Calculated using the Stuart–Maxwell test for marginal homogeneity.

**Table 3:** LATCH scores according to exposure or control group at discharge.

	LATCH Score Median (IQR)	Effect Size (r) *	*p*-Value
Control group Median (IQR)	7 (IQR 5–8)	-	-
Exposure ERAS group Median (IQR)	8 (IQR 7–9)	0.34	0.0005

IQR: Interquartile range. * Effect size (r) was calculated from the standardized Z statistic derived from the Mann–Whitney test using the formula r = Z/√N.

**Table 4:** Multivariable logistic regression analysis of factors associated with effective breastfeeding at discharge.

Variable	Β	Adjusted OR	(95% CI)	*p*-Value
Early skin-to-skin contact	0.984	2.68	1.13–6.36	0.026
Prenatal intention to breastfeed	−0.365	0.69	0.21–2.30	0.550
Parity	0.140	1.15	0.67–1.97	0.610
Previous cesarean section	−0.352	0.70	0.28–1.75	0.450
Maternal age	0.023	1.02	0.95–1.10	0.549
Educational level (university vs. secondary)	0.376	1.46	0.65–3.27	0.364
Breastfeeding support in recovery	1.000	2.72	1.02–7.22	0.045
ERAS group	1.331	3.79	1.50–9.55	0.005
Neonatal ICU admission	0.272	1.31	0.29–6.05	0.727

OR: odds ratio; CI: confidence interval; ICU: intensive care unit.

## Data Availability

The datasets generated and analyzed during the current study are available from the corresponding author upon reasonable request. No public database was used. There are no restrictions on material or data availability.

## Supplementary Materials

The following supporting information can be downloaded at: https://www.mdpi.com/article/10.3390/nursrep16040134/s1, Supplementary Table S1: STROBE Statement and ERAS components related to breastfeeding; Supplementary Table S2: ERAS components related to breastfeeding.

## Public Involvement Statement

No public involvement in any aspect of this research.

## Guidelines and Standards Statement

This study adheres to the Strengthening the Reporting of Observational Studies in Epidemiology (STROBE).

## Abbreviations

The following abbreviations are used in this manuscript:

CI	Confidence Interval
ERAS	Enhanced Recovery After Surgery
ICU	Intensive Care Unit
TAP	Transversus Abdominis Plane
IQR	Interquartile Range
OR	Odds Ratio
SD	Standard Deviation
WHO	World Health Organization
