# Supplementary material for: Breastfeeding Outcomes After Scheduled Cesarean Section Under an ERAS Pathway: An Analytical Observational Study
Source: Nurs Rep. 2026 Apr 13;16(4):134. doi: 10.3390/nursrep16040134 (PMC13118884; doi:10.3390/nursrep16040134)
Supplement: Supplementary file 1 [file nursrep-16-00134-s001.zip › nursrep-4211652-Table S2.pdf]

Table S2. ERAS components related to breastfeeding

Domain	ERAS Group	Standard Care Group	Mandatory in ERAS	Measured in Study	Clinical Notes
**Skin-to-skin contact**	Initiated early (in operating room or recovery) and encouraged continuously	Performed according to routine practice, not protocolized	Yes	Yes	Interrupted only in case of maternal or neonatal instability
**Rooming-in**	Promoted immediately after birth and maintained throughout hospitalization	Standard practice, but not actively reinforced	Yes	Yes	Separation avoided unless clinically indicated
**Early breastfeeding initiation**	Actively supported within the first hour when feasible	Variable, depending on staff and clinical context	Yes	Yes	Encouraged by midwives trained in ERAS
**Lactation support**	Proactive and continuous support from midwives trained in ERAS principles	Standard postpartum support	Yes	Yes	No fixed time schedule; support adapted to patient needs
**Supplemental feeding policy**	Not routinely used; only if clinically indicated	According to standard clinical practice	Yes	No	Indications based on neonatal or maternal criteria

Domain	ERAS Group	Standard Care Group	Mandatory in ERAS	Measured in Study	Clinical Notes
**Mother–infant separation**	Actively minimized	Occasional depending on clinical workflow	Yes	Yes	Avoided unless medically necessary
**Maternal mobilization**	Early mobilization encouraged	Standard mobilization practices	Yes	Yes	May indirectly support breastfeeding
**Analgesia (opioid-sparing)**	Multimodal, opioid-sparing strategy	Standard analgesia	Yes	Yes	Aimed to improve maternal comfort and interaction
